# Supplementary material for: Rft1 catalyzes lipid-linked oligosaccharide translocation across the ER membrane
Source: Nat Commun. 2024 Jun 17;15:5157. doi: 10.1038/s41467-024-48999-3 (PMC11182771; doi:10.1038/s41467-024-48999-3)
Supplement: Supplementary file 1 — Supplementary Information [file 41467_2024_48999_MOESM1_ESM.pdf]

Supplementary Information

Rft1 is necessary and sufficient for ER lipid-linked oligosaccharide translocation

Shuai Chen[1,2,3], Cai-Xia Pei[4,5], Si Xu[1], Hanjie Li[1], Yi-Shi liu[1], Yicheng Wang[2,3], Cheng Jin[4,*], Neta Dean[6,*] and Xiao-Dong Gao[1,2,3,*]

[1]Key Laboratory of Carbohydrate Chemistry and Biotechnology, Ministry of Education, School of Biotechnology, Jiangnan University, Wuxi 214122, China

[2]State Key Laboratory of Biochemical Engineering, Institute of Process Engineering, Chinese Academy of Sciences, Beijing 100190, China

[3]Key Laboratory of Biopharmaceutical Preparation and Delivery, Chinese Academy of Sciences, Beijing 100190, China

[4]State Key Laboratory of Mycology, Institute of Microbiology, Chinese Academy of Sciences, Beijing, China

[5]University of Chinese Academy of Sciences, Beijing, China

[6]Department of Biochemistry and Cell Biology, Stony Brook University, Stony Brook, New York 11794-5215, USA

Supplementary Figures

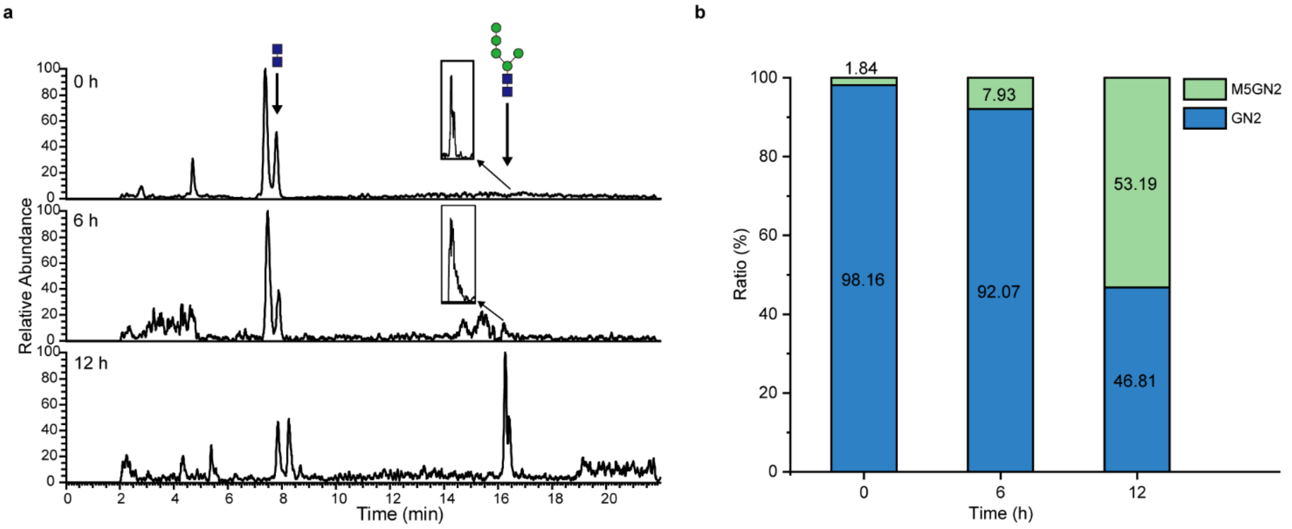

Supplementary Figure 1. Data supporting Fig. 1 | The inhibition of *RFT1* leads to the accumulation of M5GN2-PP-Dol

a UPLC chromatograms of oligosaccharides released from the LLO intermediates in *GALpr-RFT1* strain. The glucose (Glc)-repressible *GALpr-RFT1* strain grown in Glc for 0, 6 or 12 h at 30 °C, respectively. The LLO intermediates were extracted and the oligosaccharides were analyzed by UPLC-MS. Major peaks corresponding to GN2 and M5GN2 are marked. **b** The accumulation of M5 is positively correlated with the inhibition time of *RFT1*. The ratio is the peak area ratio in the UPLC chromatograms corresponding **a**.

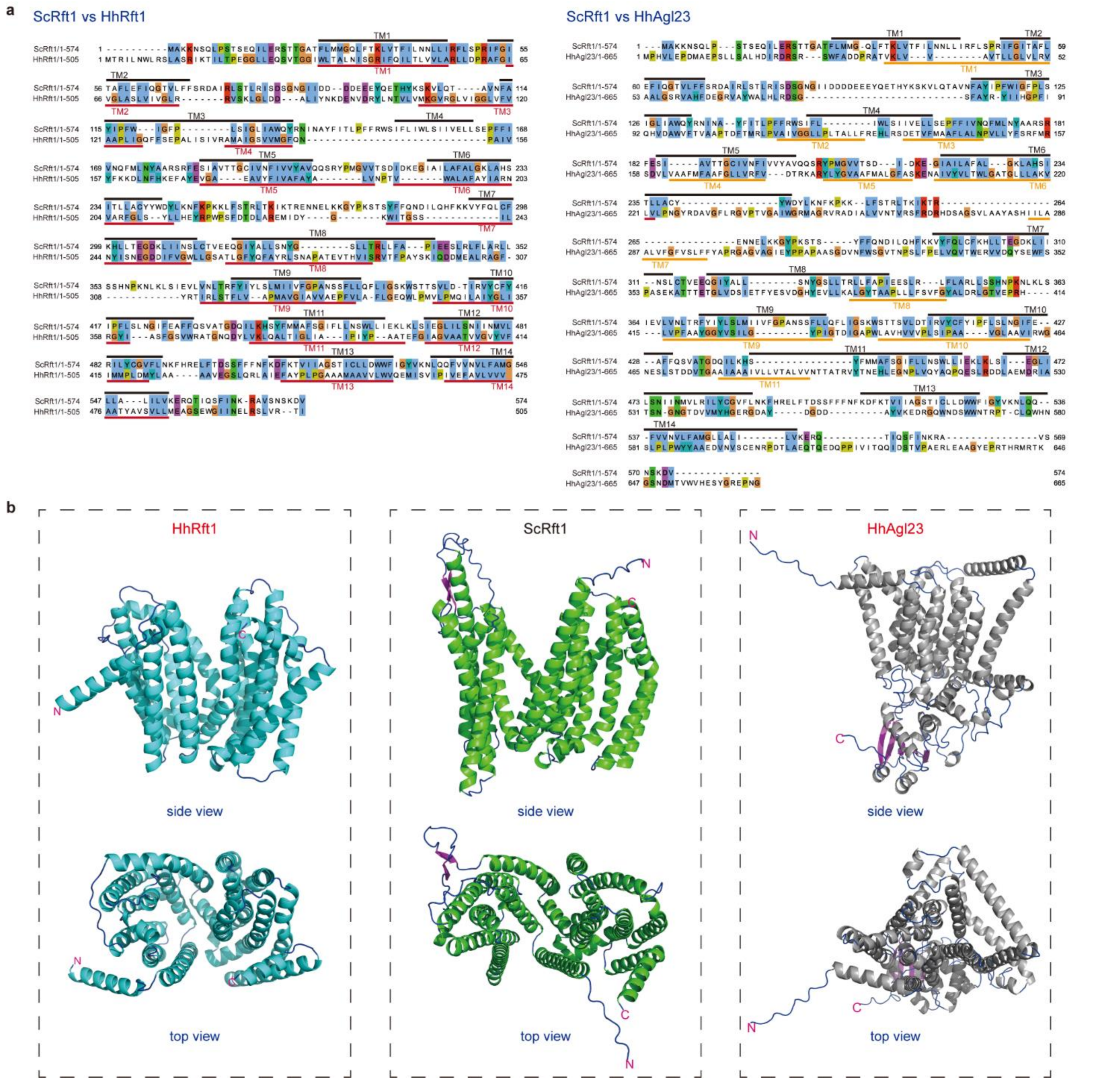

Supplementary Fig. 2. Data supporting Fig. 1 | Sequence alignment and AlphaFold prediced structure of yeast Rft1, *H. hispanica* Rft1 and Agl23 proteins

a Sequence alignment of ScRft1, HhRft1 and HhAgl23. The TM domains are indicated in black for ScRft1, red for HhRft1 and orange for HhAgl23. Sequences are from Uniprot; ScRft1 (P38206); HhRft1 (V5TSQ3) and HhAgL23 (G0HVI9). The sequence alignment was generated using Clustal Omega program and colored by the Clustal style in the Jalview. **b** Three-dimensional structural models of ScRft1, HhRft1 and HhAgl23 predicted by AlphaFold.

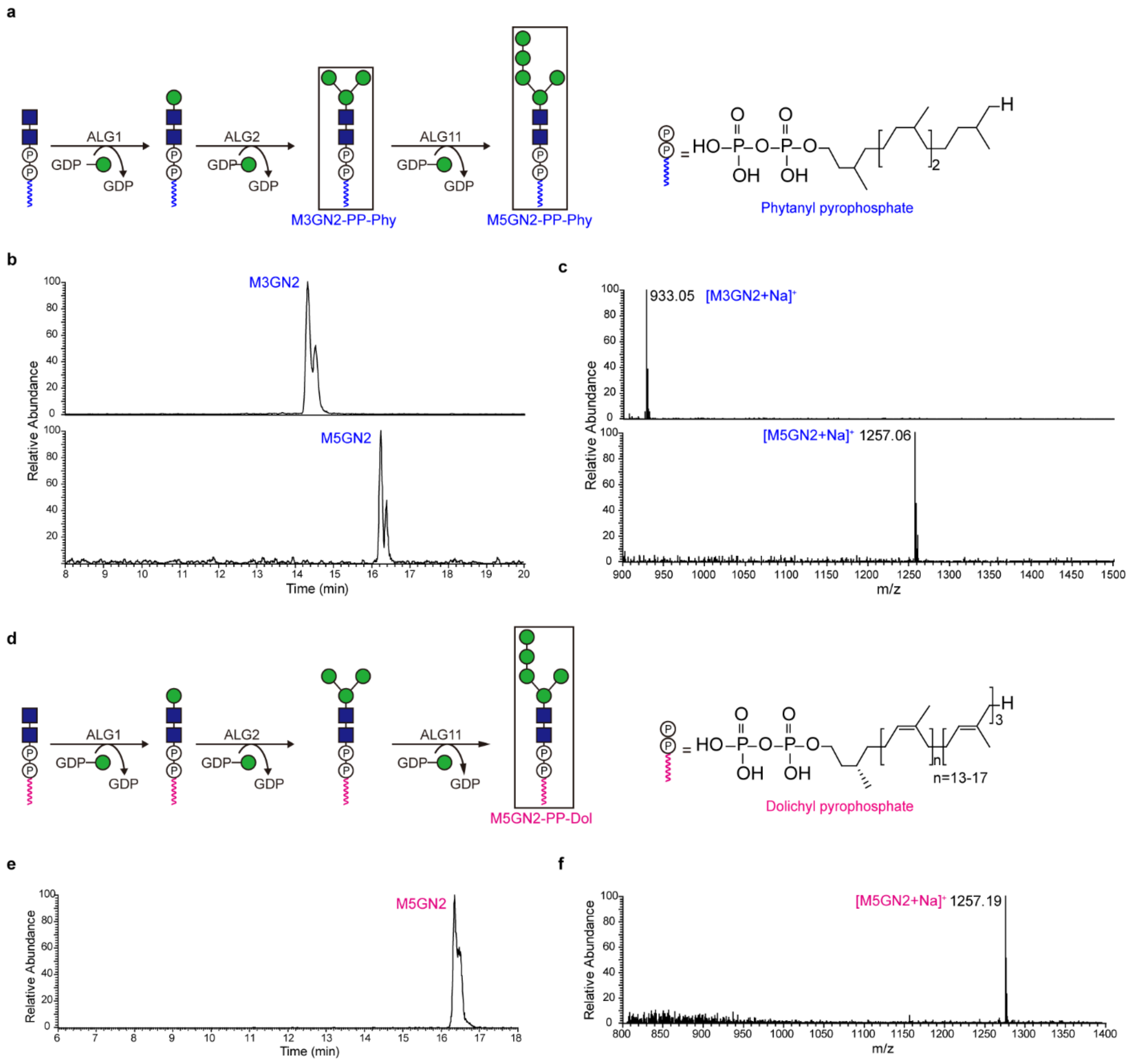

Supplementary Figure 3. Data supporting Fig. 2 | *In vitro* synthesis of M3GN2-PP-Phy, M5GN2-PP-Phy and M5GN2-PP-Dol

a Scheme for enzymatic synthesis of M3GN2-PP-Phy and M5GN2-PP-Phy. **b** UPLC chromatograms of M3GN2 and M5GN2 released from M3GN2-PP-Phy and M5GN2-PP-Phy corresponding to **a**. **c** ESI-MS spectra of M3GN2 and M5GN2 released from M3GN2-PP-Phy and M5GN2-PP-Phy corresponding to **b**. **d** Scheme for enzymatic synthesis of M5GN2-PP-Dol. **e** UPLC chromatograms of M5GN2 released from M5GN2-PP-Dol corresponding to **d**. **f** ESI-MS spectra of the M5GN2 released from M5GN2-PP-Dol corresponding to **d**.

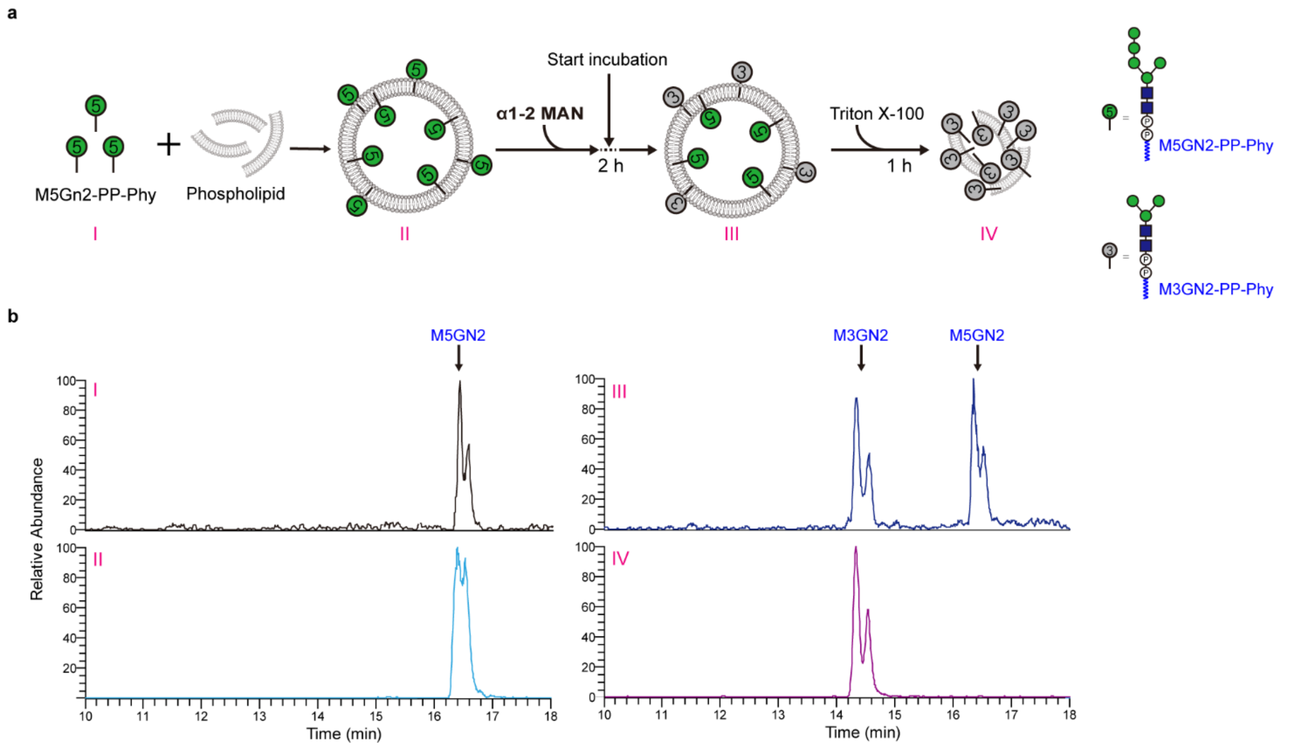

Supplementary Figure 4. Data supporting Fig. 2 | Validation of α1-2 mannosidase-based flippase assay

a Schematic diagram of liposome construction. I: M5GN2-PP-Phy and the mixture of phosphatidyl-choline (PC) and phosphatidyl-serine (PS). II: Liposomes were generated by the treatment with SM-2 Bio-Beads. III: M5GN2-PP-Phy in the outer leaflet of liposomes were digested with α1-2 mannosidase at 30 °C for 2 h to generate M3GN2-PP-Phy. IV: Liposomes permeabilized with 1% Triton X-100 for 1 h results in complete mannosidase digestion of all M5GN2-PP-Phy to generate M3GN2-PP-Phy. **b** UPLC chromatograms of M3GN2 and M5GN2 released from M3GN2-PP-Phy and M5GN2-PP-Phy of different steps corresponding to **a**.

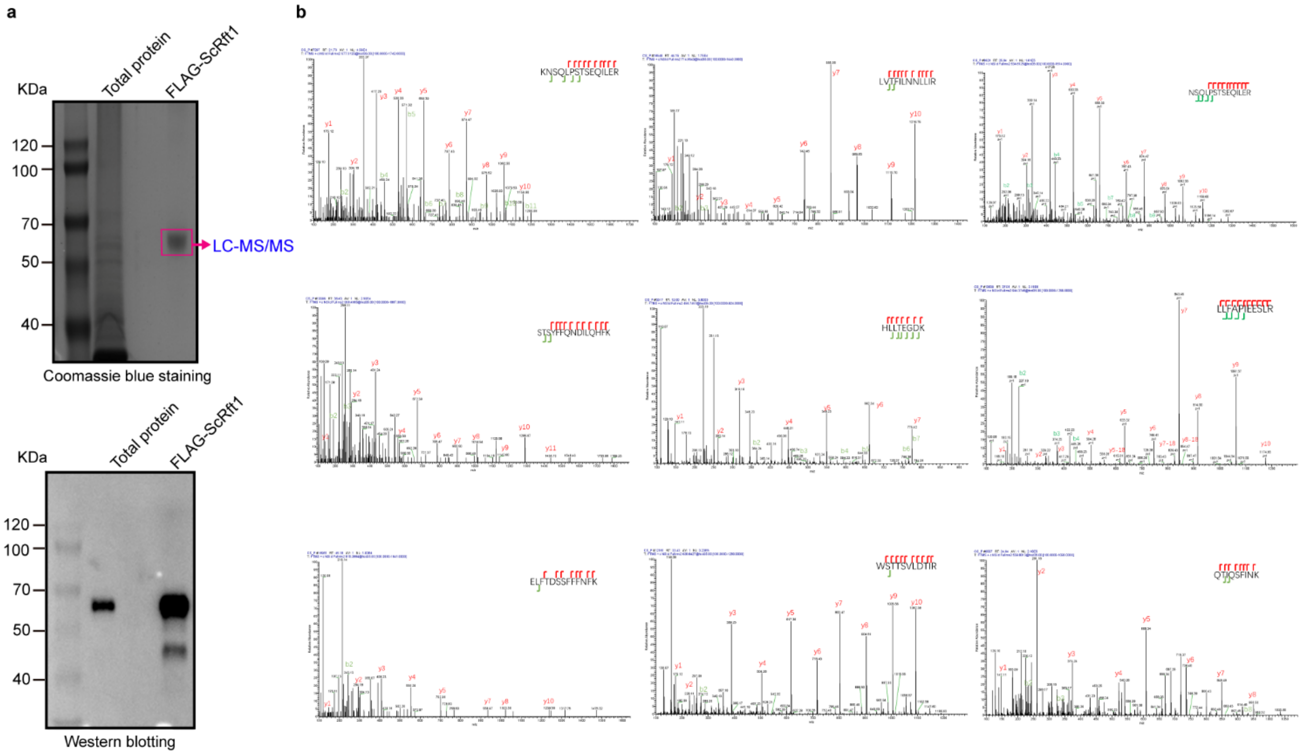

c

FLAG-ScRft1:

MDYKDDDDKAKKNSQLPSTSEQILERSTTGATFLMMGQLFTKLVTFILNNLLIRFLSPRIFGITAFLEFIQGTVLFFSRDAIRLSTLRISDSGNGIIDDDDEEEYQETHYKSKVLQTA
VNFAYIPFWIGFPLSIGLIAWQYRNINAYFITLPFFRWSIFLIWLSIIVELLSEPFFIVNQFMLNYAARSRFESIAVTTGCIVNFIVVYAVQQSRYPMGVVTSDIDKEGIAILAFALGKLA
HSITLLACYYWDYLKNFKPKKLFSTRLTKIKTRENNELKKGYPKSTSYFFQNDILQHFKKVYFQLCFKHLLTEGDKLIINSLCTVEEQGIYALLSNYGSLLTRLLFAPIEESLRLFLA
RLLSSHNPKNLKLSIEVLVNLTRFYIYLSLMIIVFGPANSSFLLQFLIGSKWSTTSVLDTIRVYCFYIPFLSLNGIFEAFFQSVATGDQILKHSYFMMAFSGIFLLNSWLLIEKLKLSIE
GLILSNIINMVLRILYCGVFLNKFHRELFTDSSFFFNFKDFKTVIIAGSTICLLDWWFIGYVKNLQQFVVNVLFAMGLLALILVKERQTIQSFINKRAVSNSKDV*

Supplementary Figure 5. Data supporting Fig. 2 | LC-MS/MS analysis of purified ScRft1

a SDS-PAGE analysis of total proteins and purified FLAG-ScRft1 (top panel) and analyzed by Western blotting with anti-FLAG antibody (bottom panel). **b** LC-MS/MS chromatograms of peptides. The gel marked in the red box was cut out, the proteins were enzymatically hydrolyzed into peptides and analyzed by LC-MS/MS. **c** Matched peptides of Rft1 shown in red.

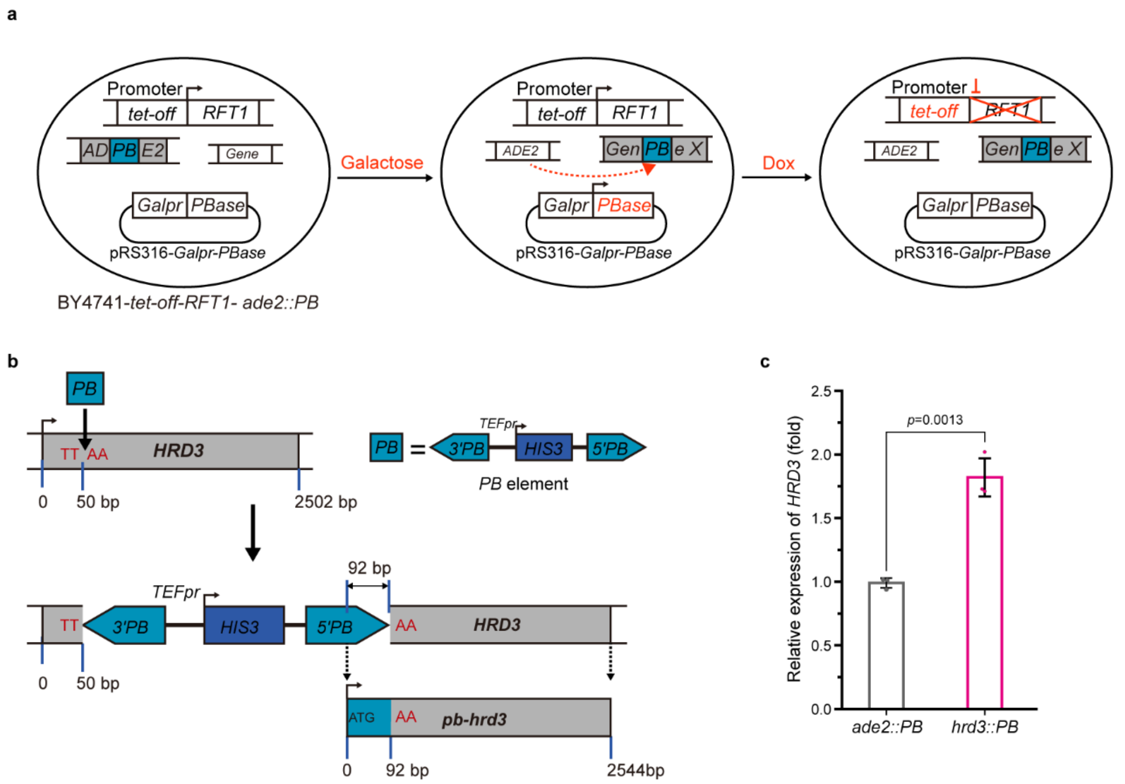

Supplementary Figure 6. Data supporting Fig. 3 | *PiggyBac (PB)* transposon-based mutagenesis and screening of *rft1*Δ suppressors

a Schematic representation of *rft1*Δ yeast suppressor screen. BY4741 was used as the parental strain to construct the $P_{tet\text{-}off}$-*RFT1 ade2::PB* strain, in which the endogenous *RFT1* promoter was replaced by a *tet-off* promoter and a *PB* transposon was inserted into a TTAA sequence in *ADE2*. A *URA3* plasmid with a *GAL1-10* promoter-driven transposase, pRS316-*GALpr-PBase,* was introduced into this strain. Induction of PBase with galactose causes excision of *PB* from *ADE2* gene and its saturating insertion into genomic TTAA targets. PB insertional mutants that suppress the inviability of doxycycline-mediated *RFT1* shutoff were isolated and the genomic sites of PB insertion characterized by sequencing. **b** A schematic of the *PB* insertion site in the *hrd3::PB* $P_{tet\text{-}off}$-*RFT1* mutant . The *PB* element was inserted into the TTAA site located at -50 bp (upstream of start codon) of *HRD3*. This insertion results in a replacement of the first 50 bp of *HRD3* by 92 bp of PB C-terminal sequence. **c** Quantitative PCR of *HRD3* mRNA levels in BY4741-$P_{tet\text{-}off}$-*RFT1- ade2::PB* and *hrd3::PB* strains. *TDH1* mRNA values were used for normalization. The bars represent RQ (relative quantification) values ± RQmax and RQmin (error bars) of triplicate samples. Data in **c** are from three independent biological repeats and *P* values are from t test.

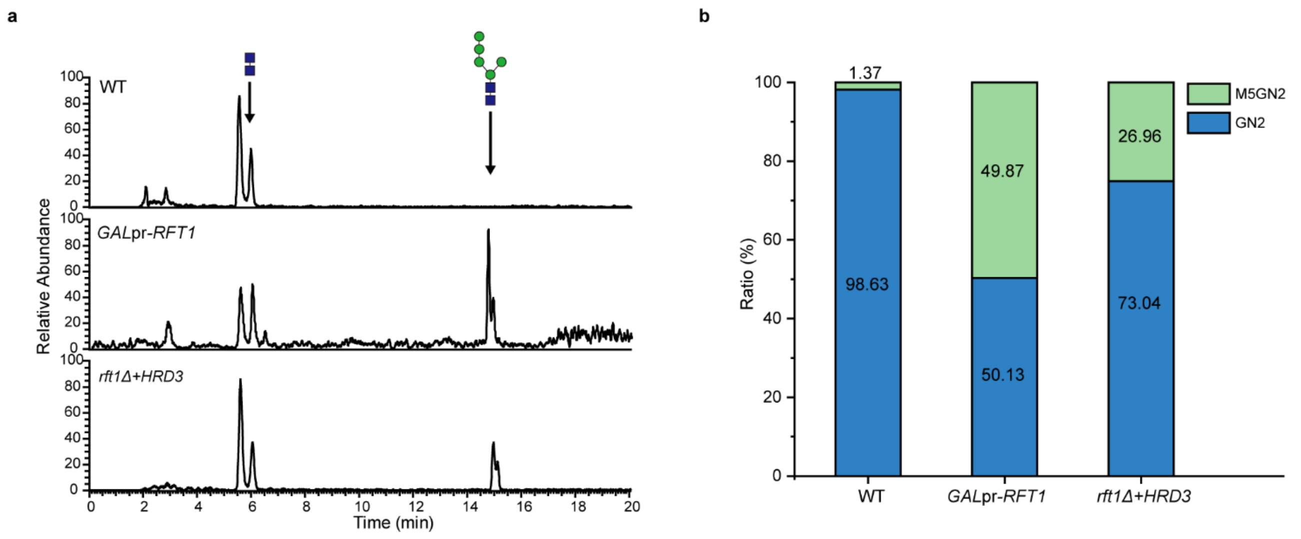

Supplementary Figure 7. Data supporting Fig. 4 | UPLC-MS measurement of M5GN2-PP-Dol in wild type, *P*$_{GAL}$*-RFT1* and *rft1*Δ+*HRD3* strains

a UPLC chromatograms of oligosaccharides released from the LLO intermediates in WT, *GALpr-RFT1* and *rft1*Δ-*HRD3* strains. The strains were grown in YPAD for 12 h at 30 °C. The LLO intermediates were extracted and the oligosaccharides were analyzed by UPLC-MS. Major peaks corresponding to GN2 and M5GN2 are marked. **b** The relative accumulation of M5 in different strains. The ratio is the peak area ratio in the UPLC chromatograms corresponding **a**.

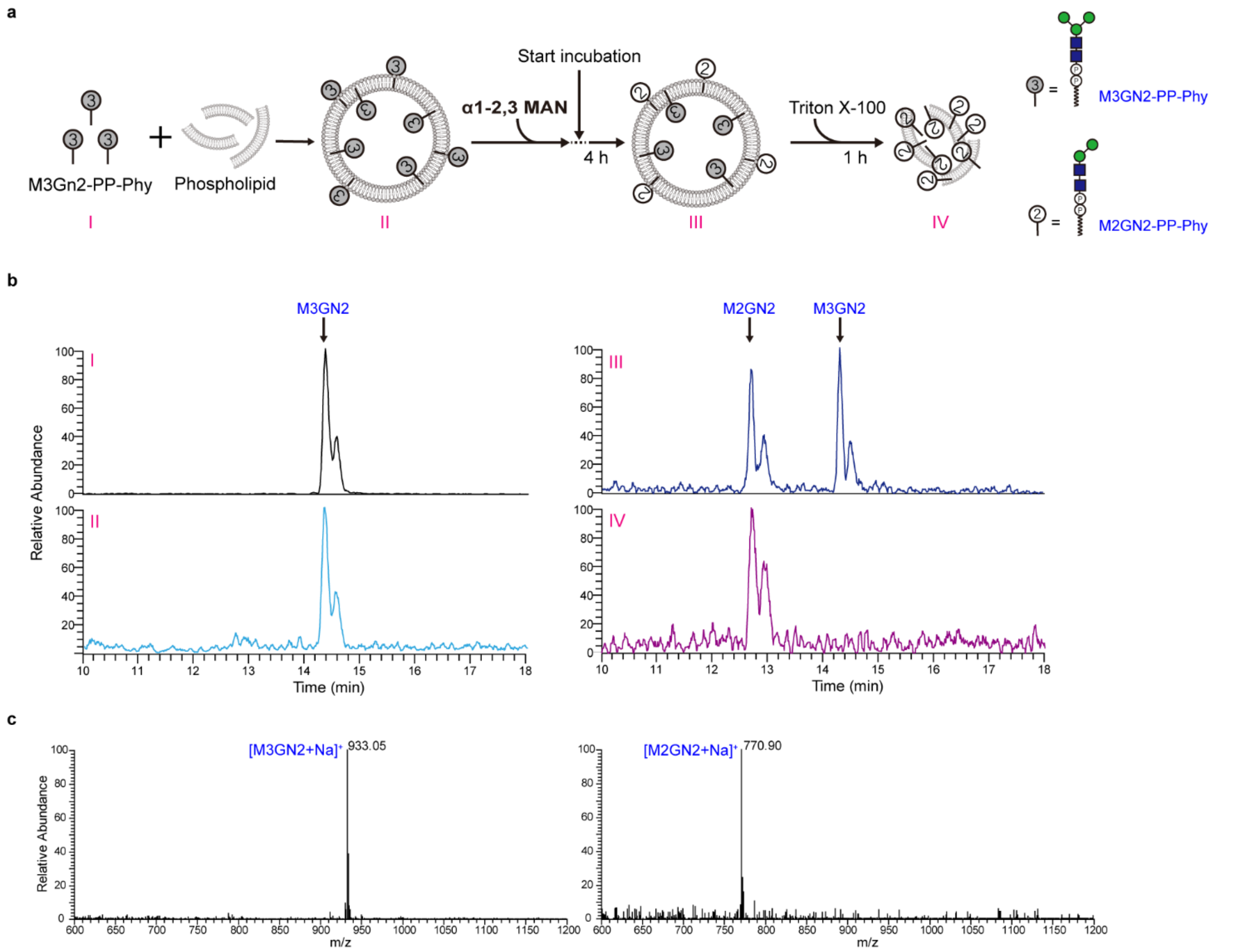

Supplementary Fig. 8. Data supporting Fig. 4 | Validation of α1-2,3 mannosidase-based flippase assay

a Schematic diagram of liposome construction. I: M3GN2-PP-Phy and the mixture of PC and PS. II: Liposomes were generated by the treatment with SM-2 Bio-Beads. III: M3GN2-PP-Phy in the outer leaflet of liposomes were digested with α1-2,3 mannosidase for 4 h to generate M2GN2-PP-Phy. IV: Liposomes permeabilized with 1% Triton X-100 for 1 h results in complete mannosidase digestion of all M3GN2-PP-Phy to generate M2GN2-PP-Phy. **b** UPLC chromatograms of M2GN2 and M3GN2 released from M2GN2-PP-Phy and M3GN2-PP-Phy of different steps corresponding to **a**. **c** ESI-MS spectra of M2GN2 and M3GN2 released from M2GN2-PP-Phy and M3GN2-PP-Phy corresponding to **b**-III.

Tables

Supplementary Table 1. Strains used in this study

Strains	Description
W303a	*MATα ade2-1 ura3-1 his3-11 trp1-1 leu2-3,112 can1-100*
W303a-*rft1Δ-ScRFT1*	W303a-*RFT1 :: kanMX4* with pRS316-GAPII-*ScRFT1*
W303a-*GALpr-RFT1*	W303a-*RFT1pr :: GAL1/10pr-kanMX4*
W303a-*rft1Δ-FLAG-ScRFT1*	W303a-*rft1Δ* with YEp351-GAPII-*FLAG-ScRFT1*
W303a-*rft1Δ-HsRFT1-FLAG*	W303a-*rft1Δ* with YEp351-GAPII-*HsRFT1-FLAG*
W303a-*rft1Δ-HRD3*	W303a-*rft1Δ* with YEp351-GAPII-*HRD3*
Haloarcula hispanica (Hh)	*H. hispanica* ATCC 33,960
Hh-CBD-*HhAGL22*	pWL-CBD-*HhAGL22* in Hh
Hh-CBD-*HhAGL23*	pWL-CBD-*HhAGL22* in Hh
BY4741	*MATa his3Δ1 leu2Δ met15Δ ura3Δ*
BY4741-*tet-off-RFT1*	As in BY4741 and *RFT1pr :: tet-off*
BY4741-*tet-off-RFT1*-PB	As in BY4741-*tet-off-RFT1-ADE2 :: 3'PB-TEFpr-HIS3–5'PB*
E. coli Rosetta-Alg1/2	pET28-Alg1/2 in Rosetta
E. coli Rosetta-Alg1/2/11	pET28-Alg1/2/11 in Rosetta
E. coli Rosetta-BT3990	pET28-BT3990 in Rosetta

[1] Gene or species names are denoted by italics.

Supplementary Table 2. Plasmids used in this study

Plasmid	Description
YEp351-GAPII	LEU2/2μ yeast shuttle vector containing TDH3 promoter
pRS316-GAPII	URA3/2μ yeast shuttle vector containing TDH3 promoter
YEp351-GAPII-*ScRFT1*	*ScRFT1* in YEp351-GAPII
YEp351-GAPII-*HsRFT1*	*HsRFT1* in YEp351-GAPII
YEp351-GAPII-*TbRFT1*	*TbRFT1* in YEp351-GAPII
YEp351-GAPII-*HhRFT1*	*HhRFT1* in YEp351-GAPII
YEp351-GAPII-*HvRFT1*	*HvRFT1* in YEp351-GAPII
YEp351-GAPII-*HhAGL23*	*HhAGL23* expressed in YEp351-GAPII
YEp351-GAPII-*HhAGL22*	*HhAGL22* in YEp351-GAPII
YEp351-GAPII-*HsCLTPM1L*	*HsCLTPM1L* in YEp351-GAPII
YEp351-GAPII-*TaMURJ*	*TaMURJ* in YEp351-GAPII
YEp351-GAPII-*FLAG-ScRFT1*	*ScRFT1* with N-terminal FLAG-tag in YEp351-GAPII
YEp351-GAPII-*HsRFT1-FLAG*	*HsRFT1* with C-terminal FLAG-tag in YEp351-GAPII
YEp351-GAPII-*HRD3*	*HRD3* expressed in YEp351-GAPII
pWL-CBD	Archaeal constitutive promoter PrP16 and cellulose-binding domain from *Clostridium thermocellum*.
pWL-CBD-*HhAGL23*	*HhAGL23* with N-terminal CBD-tag in pWL- CBD
pWL-CBD-*HhAGL22*	*HhAGL22* with N-terminal CBD-tag in pWL- CBD
pET28	T7 promoter with His6 tag at N-terminal
pET28-Alg1/2	*ScALG1* and *ScALG2* co-expressed in pET28
pET28-Alg1/2/11	*ScALG1, ScALG2* and *ScALG11* co-expressed in pET28
pET28-BT3990	Alpha1-2 mannosidase from *Bacteroides thetaiotaomicron* in pET28
pRS316-GALpr-*PBase*	*PBase* in pRS316 containing GAL1 promoter
