# Supplementary material for: Rft1 catalyzes lipid-linked oligosaccharide translocation across the ER membrane
Source: Nat Commun. 2024 Jun 17;15:5157. doi: 10.1038/s41467-024-48999-3 (PMC11182771; doi:10.1038/s41467-024-48999-3)
Supplement: Supplementary file 3 — Reporting Summary [file 41467_2024_48999_MOESM3_ESM.pdf]

nature portfolio

Corresponding author(s): Xiao-Dong Gao

Last updated by author(s): Mar 5, 2024

Reporting Summary

Nature Portfolio wishes to improve the reproducibility of the work that we publish. This form provides structure for consistency and transparency in reporting. For further information on Nature Portfolio policies, see our Editorial Policies and the Editorial Policy Checklist.

Statistics

For all statistical analyses, confirm that the following items are present in the figure legend, table legend, main text, or Methods section.

n/a	Confirmed	
☐	☒	The exact sample size (*n*) for each experimental group/condition, given as a discrete number and unit of measurement
☐	☒	A statement on whether measurements were taken from distinct samples or whether the same sample was measured repeatedly
☐	☒	The statistical test(s) used AND whether they are one- or two-sided<br>*Only common tests should be described solely by name; describe more complex techniques in the Methods section.*
☒	☐	A description of all covariates tested
☐	☒	A description of any assumptions or corrections, such as tests of normality and adjustment for multiple comparisons
☐	☒	A full description of the statistical parameters including central tendency (e.g. means) or other basic estimates (e.g. regression coefficient) AND variation (e.g. standard deviation) or associated estimates of uncertainty (e.g. confidence intervals)
☐	☒	For null hypothesis testing, the test statistic (e.g. *F*, *t*, *r*) with confidence intervals, effect sizes, degrees of freedom and *P* value noted<br>*Give P values as exact values whenever suitable.*
☒	☐	For Bayesian analysis, information on the choice of priors and Markov chain Monte Carlo settings
☒	☐	For hierarchical and complex designs, identification of the appropriate level for tests and full reporting of outcomes
☐	☒	Estimates of effect sizes (e.g. Cohen's *d*, Pearson's *r*), indicating how they were calculated

Our web collection on statistics for biologists contains articles on many of the points above.

Software and code

Policy information about availability of computer code

Data collection	Tianneng imaging system AIIDOC ALL software was used to collect Western blot and SDS PAGE data; Thermo Fisher Scientific SmartStatus software was used to collect data of UPLCM analyses.
Data analysis	OriginPro 2021 was used for statistical analysis and graph output; Thermo Xcalibur Qual Browser 2.2 SP1.48 was used for quantitative analysis of UPLC-MS data; Clustal Omega program was usde for sequence alignment; Open-Source PyMOL 2.3 was used for figure output of the predicted protein structure.

For manuscripts utilizing custom algorithms or software that are central to the research but not yet described in published literature, software must be made available to editors and reviewers. We strongly encourage code deposition in a community repository (e.g. GitHub). See the Nature Portfolio guidelines for submitting code & software for further information.

Data

Policy information about availability of data

All manuscripts must include a data availability statement. This statement should provide the following information, where applicable:

- Accession codes, unique identifiers, or web links for publicly available datasets
- A description of any restrictions on data availability
- For clinical datasets or third party data, please ensure that the statement adheres to our policy

All data are included in the Article and its supplementary Information. Source data are provided with this paper.

Research involving human participants, their data, or biological material

Policy information about studies with human participants or human data. See also policy information about sex, gender (identity/presentation), and sexual orientation and race, ethnicity and racism.

Reporting on sex and gender	n/a
Reporting on race, ethnicity, or other socially relevant groupings	n/a
Population characteristics	n/a
Recruitment	n/a
Ethics oversight	n/a

Note that full information on the approval of the study protocol must also be provided in the manuscript.

Field-specific reporting

Please select the one below that is the best fit for your research. If you are not sure, read the appropriate sections before making your selection.

☒ Life sciences ☐ Behavioural & social sciences ☐ Ecological, evolutionary & environmental sciences

For a reference copy of the document with all sections, see nature.com/documents/nr-reporting-summary-flat.pdf

Life sciences study design

All studies must disclose on these points even when the disclosure is negative.

Sample size	No statistical method was used to predetermine the sample size. Typically, for knockout genes or validation of genes by knockout, multiple (2-5) cell clones were obtained and confirmed for knockout by PCR, Western blotting. One clone from the most representative clones was chosen for experiments
Data exclusions	No data were excluded from the analysis.
Replication	All experiments shown in the manuscript were reproducible. Number of independent biological repeats are stated in the figure legends. All attempts at replication were successful.
Randomization	This paper does not include comparison studies among groups, so this issue does not apply.
Blinding	No blinding was applied.

Reporting for specific materials, systems and methods

We require information from authors about some types of materials, experimental systems and methods used in many studies. Here, indicate whether each material, system or method listed is relevant to your study. If you are not sure if a list item applies to your research, read the appropriate section before selecting a response.

Materials & experimental systems

n/a	Involved in the study	
☐	☒	Antibodies
☐	☒	Eukaryotic cell lines
☒	☐	Palaeontology and archaeology
☒	☐	Animals and other organisms
☒	☐	Clinical data
☒	☐	Dual use research of concern
☒	☐	Plants

Methods

n/a	Involved in the study	
☒	☐	ChIP-seq
☒	☐	Flow cytometry
☒	☐	MRI-based neuroimaging

Antibodies

Antibodies used	The following antibodies were used in this study: CPY (ab113685, Abcam), DYKDDDDK (FLAG) (# HT201-01, TransGen Biotech)

Antibodies used	(ab113685, Abcam).
Validation	All antibodies used in our study have been validated. Detailed information could be found on the manufactures' website as listed below.<br>PY (ab113685, Abcam), https://www.abcam.cn/products/primary-antibodies/carboxypeptidase-y-antibody-10a5b5-ab113685.html;<br>DYKDDDDK (FLAG) (# HT201-01, TransGen Biotech), https://www.transgen.com/antibody_tag/371.html.

Eukaryotic cell lines

Policy information about cell lines and Sex and Gender in Research

Cell line source(s)	Saccharomyces cerevisiae (W303a, BY4741), Haloarcula hispanica (ATCC 33,960), E. coli (Rosetta) were used in this study.
Authentication	None of the cell lines used were authenticated.
Mycoplasma contamination	All parent cells tested negative for mycoplasma contamination, knockout cells were not tested for mycoplasma contamination.
Commonly misidentified lines<br>(See ICLAC register)	None of the cell lines used are listed in the ICLAC database.

Plants

Seed stocks	n/a
Novel plant genotypes	n/a
Authentication	n/a
